# Supplementary material for: Loss of mTORC1 signalling impairs β-cell homeostasis and insulin processing
Source: Nat Commun. 2017 Jul 12;8:16014. doi: 10.1038/ncomms16014 (PMC5510183; doi:10.1038/ncomms16014)
Supplement: Supplementary Information [file ncomms16014-s1.pdf]

Type of file: PDF
Size of file: 0 KB
Title of file for HTML: Supplementary Information
Description: Supplementary figures and supplementary tables.

Type of file: PDF
Size of file: 0 KB
Title of file for HTML: Peer review file
Description:

Supplementary Figure 1

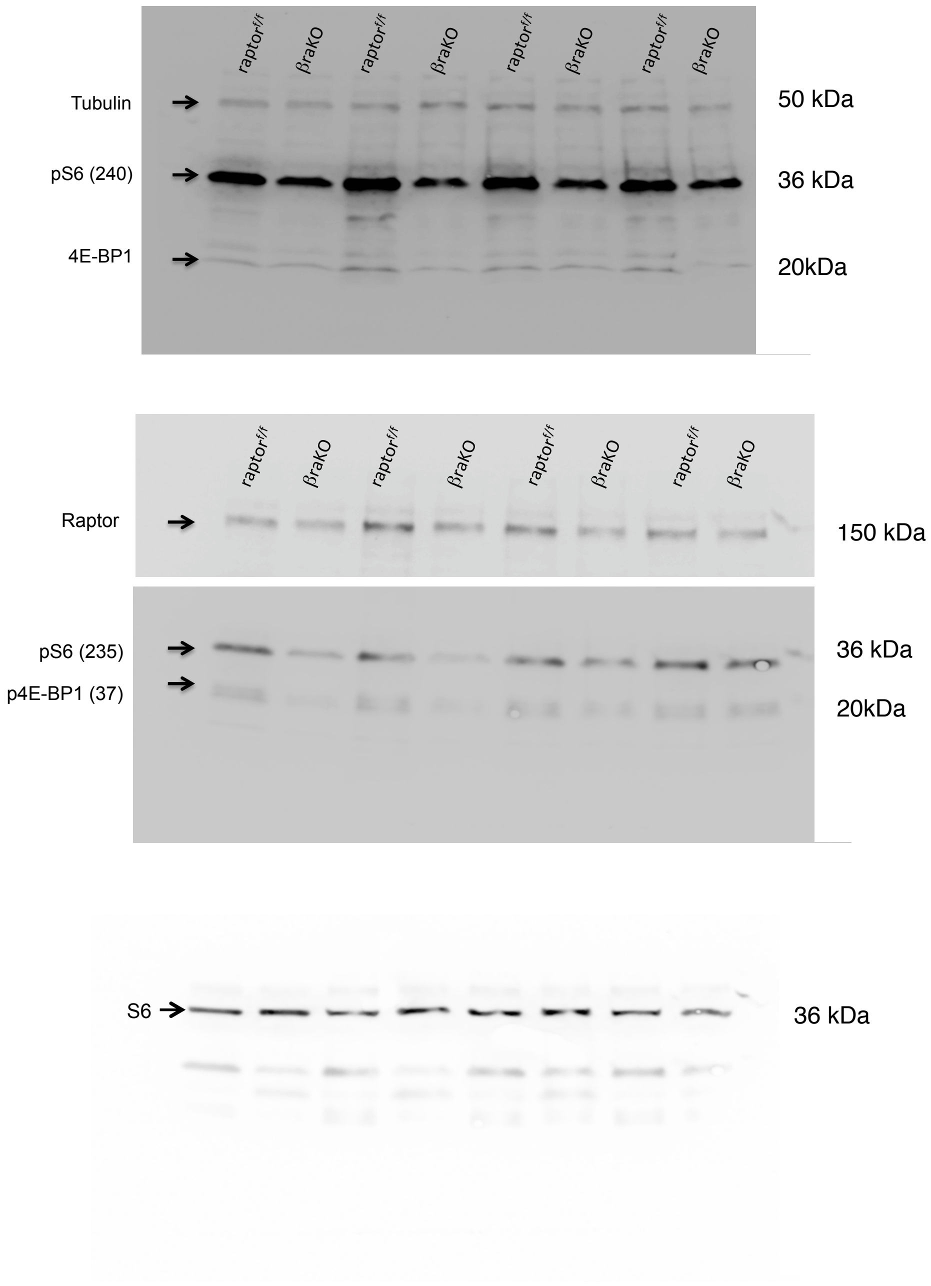

Supplementary Figure 1: Full western blots of Raptor, tubulin, pS6 (240), pS6 (235), p4E-BP1 and 4E-BP1 in *raptor*$^{f/f}$ and *βraKO* mice.

Three membranes with samples from the same mice were used for Figure 1a. Different antibodies and time exposure were used to obtain the images in the figure. Membranes were cut around 100 kDa before probing with antibodies to increase the number of antibodies used per membrane.

Supplementary Figure 2

a

60 Days

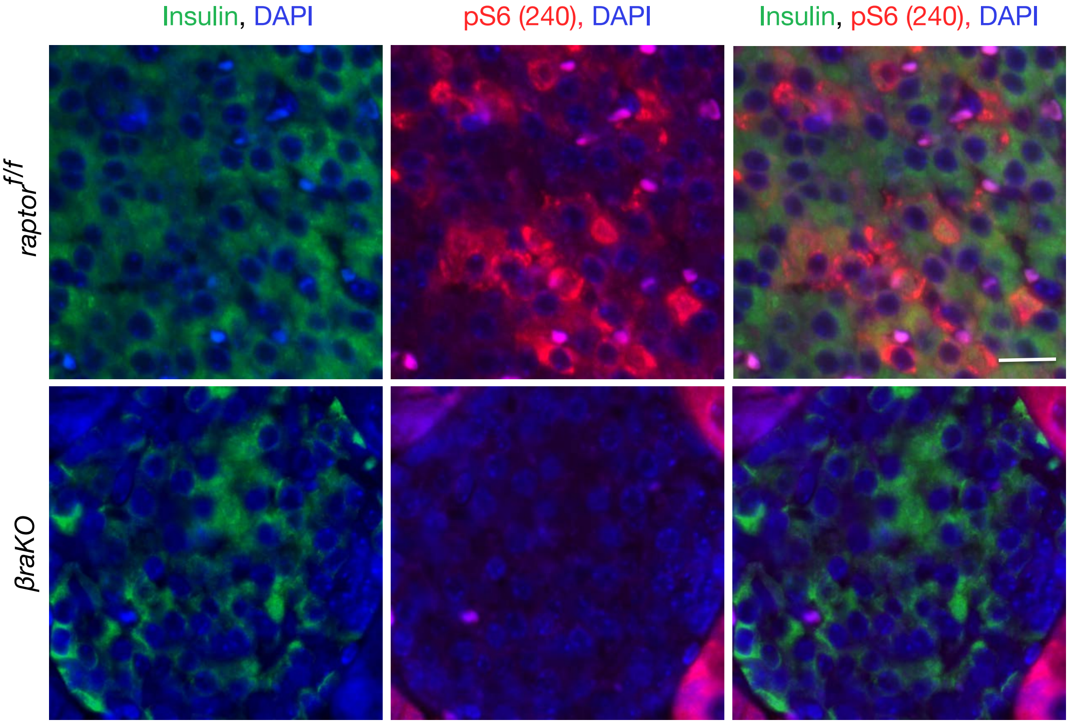

b

30 days

βraKO; CAG-GFP

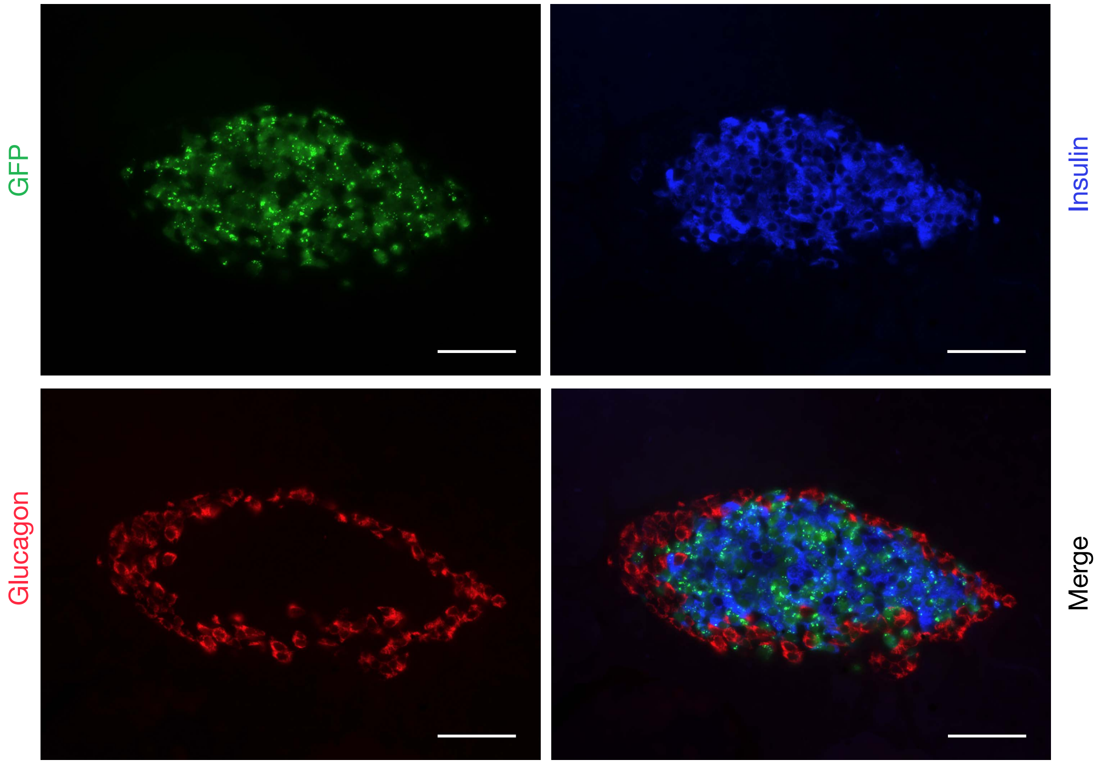

Supplementary Figure 2. Assessment of mTORC1 activity by S6 phosphorylation and recombination efficiency in *βraKO*; *CAG-GFP* reporter mouse.

(a) Immunostaining for insulin (green), pS6 (S240) (red) and DAPI (blue) in *raptor*$^{f/f}$ and *βraKO* mice at 60 days of age. Scale bars, 20 μM. Images are representative of 3 mice. (b) Immunostaining for glucagon (red), insulin (blue) and GFP (green) in pancreas from 30 day-old *βraKO; CAG-GFP* mouse. Scale bar 50 μM. Image is representative of 3 mice.

Supplementary Figure 3

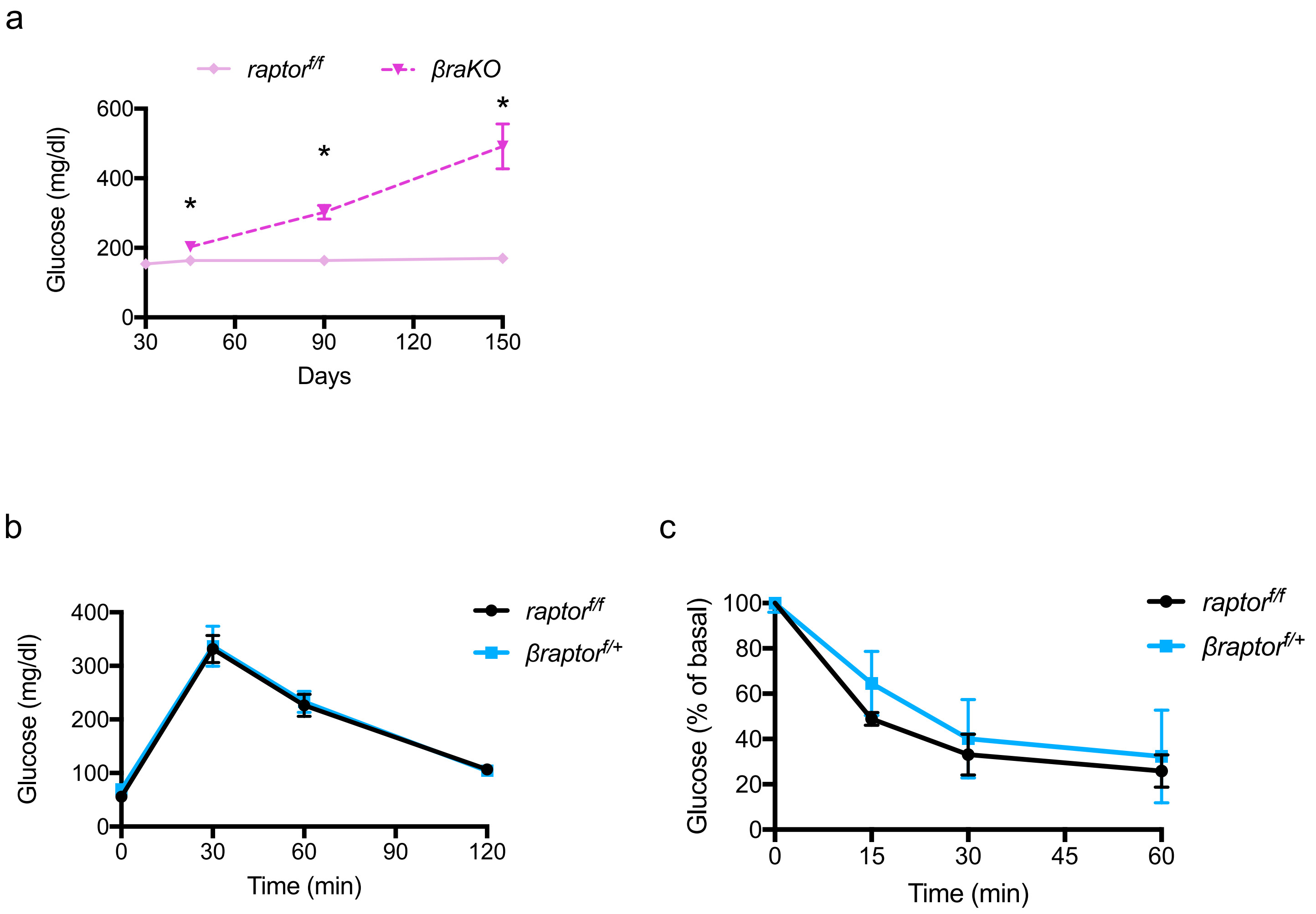

Supplementary Figure 3. Fed glucose levels in control and *βraKO* females and glucose tolerance and insulin sensitivity in raptor heterozygous mice.

(a) Random blood glucose levels in female $raptor^{f/f}$ and *βraKO* mice during the first 150 days of age (n=10). (b) Intraperitoneal glucose tolerance test in $raptor^{f/f}$ and $\beta raptor^{f/+}$ at 60 days of age (n=5). (c) Insulin tolerance test (ITT) in $raptor^{f/f}$ and $\beta raptor^{f/+}$ at 60 days of age. Data are shown as means ± s.e.m, $*P < 0.05$; Nonparametric U test (Mann-Whitney).

Supplementary Figure 4

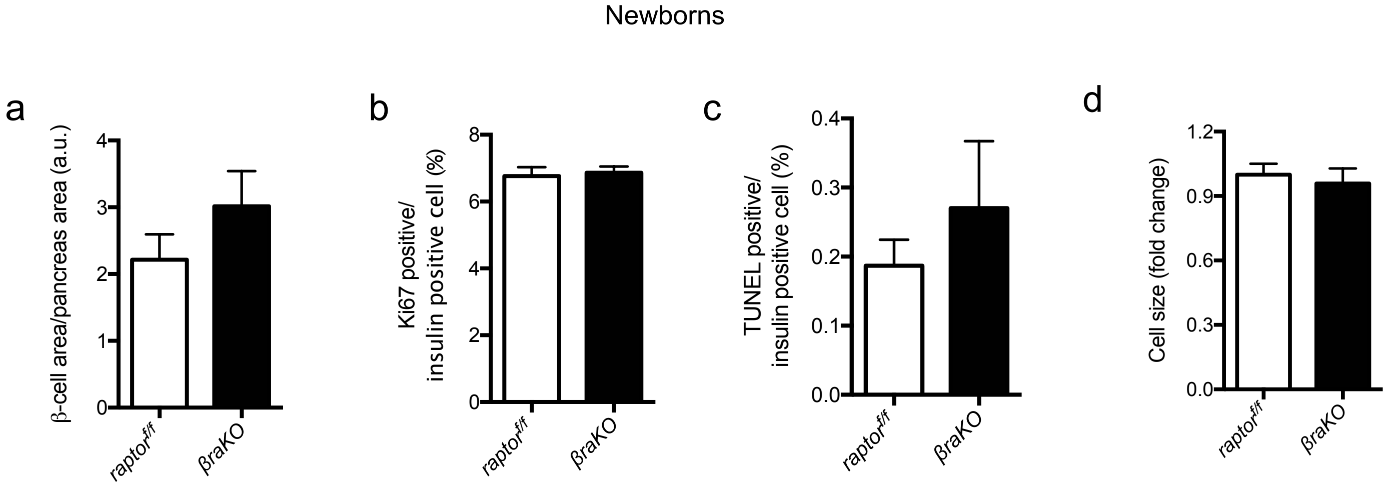

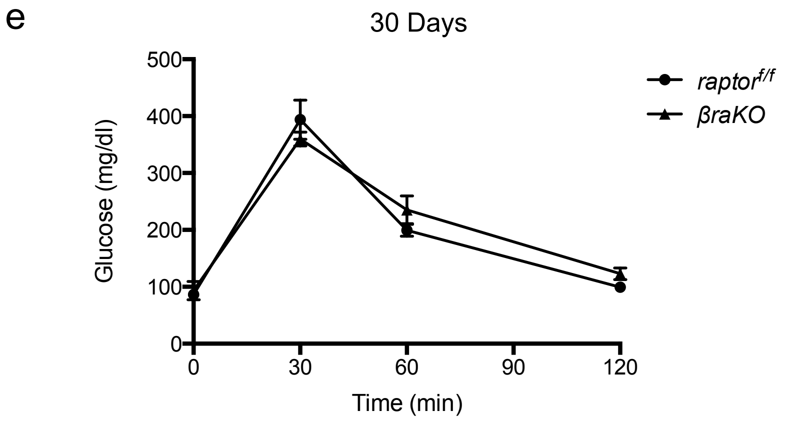

Supplementary Figure 4. Analysis of morphology in newborns and glucose tolerance at 30 days of age.

(a) Assessment of β-cell area in control and *βraKO* mice during the first 24 hours of life (neonates) (n=6). (b) β-cell proliferation in sections from control and *βraKO* neonates stained for Ki67 and insulin (n=6). (c) TUNEL assay in sections from control and *βraKO* neonates stained (n=6). (d) Measure of cell size expressed as a fold change in the same group of mice (n=6). (e) Intraperitoneal glucose tolerance test in 30 day-old mice (n=5). Data expressed as means ± s.e.m., $*P < 0.05$. Nonparametric U test (Mann-Whitney).

Supplementary Figure 5

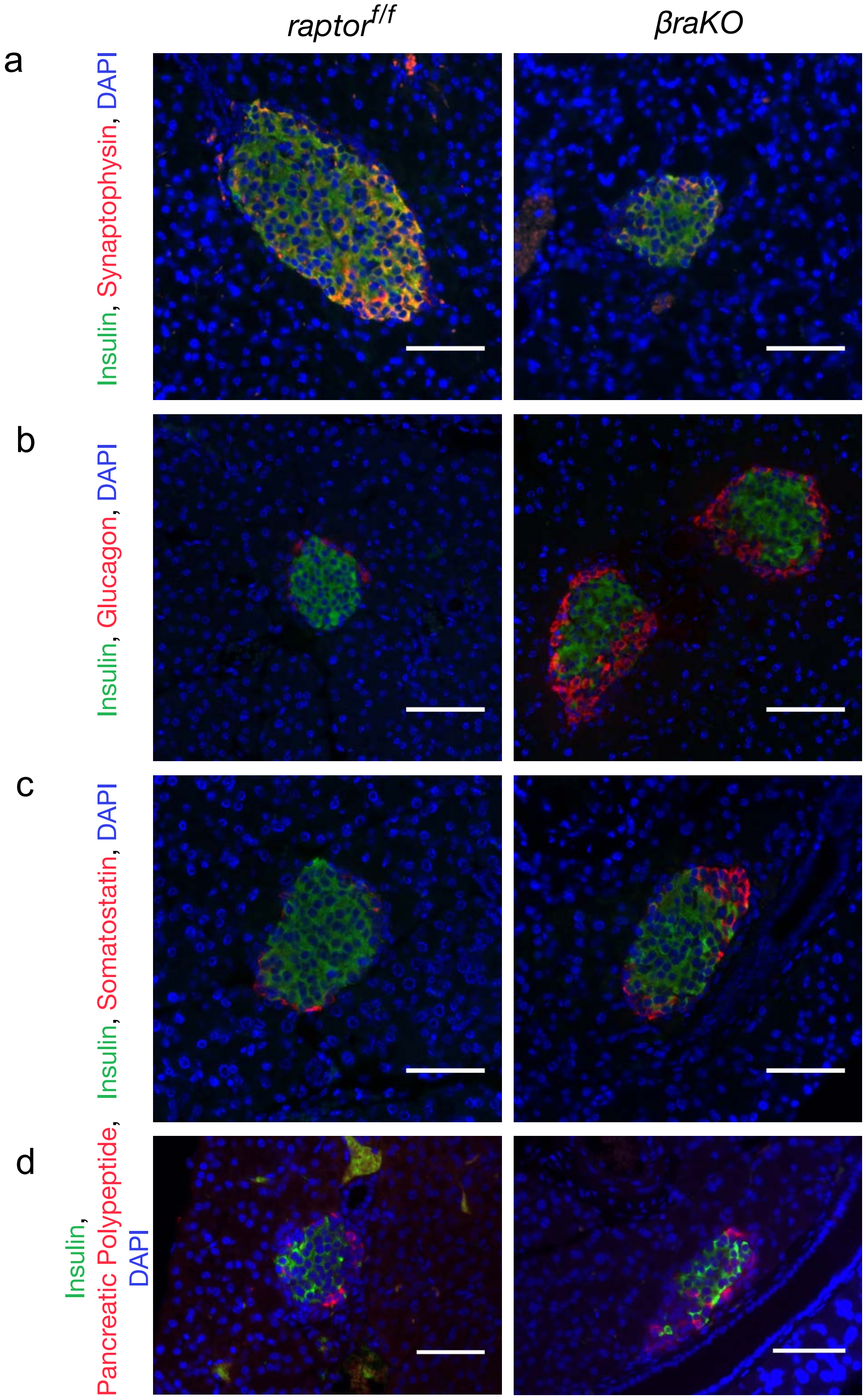

Supplementary Figure 5. Assessment of different endocrine cells in sections from control and *βraKO* mice at 30 days of age.

Immunostaining for insulin (green), (a) synaptophysin (red), (b) glucagon (red), (c) somatostatin (red) (d) and pancreatic polypeptide (red) and counterstained with DAPI (blue) in *raptor*$^{f/f}$ and *βraKO* mice at 30 days of age. Scale bar 50 μM. Images are representative of 3 mice per group.

Supplementary Figure 6

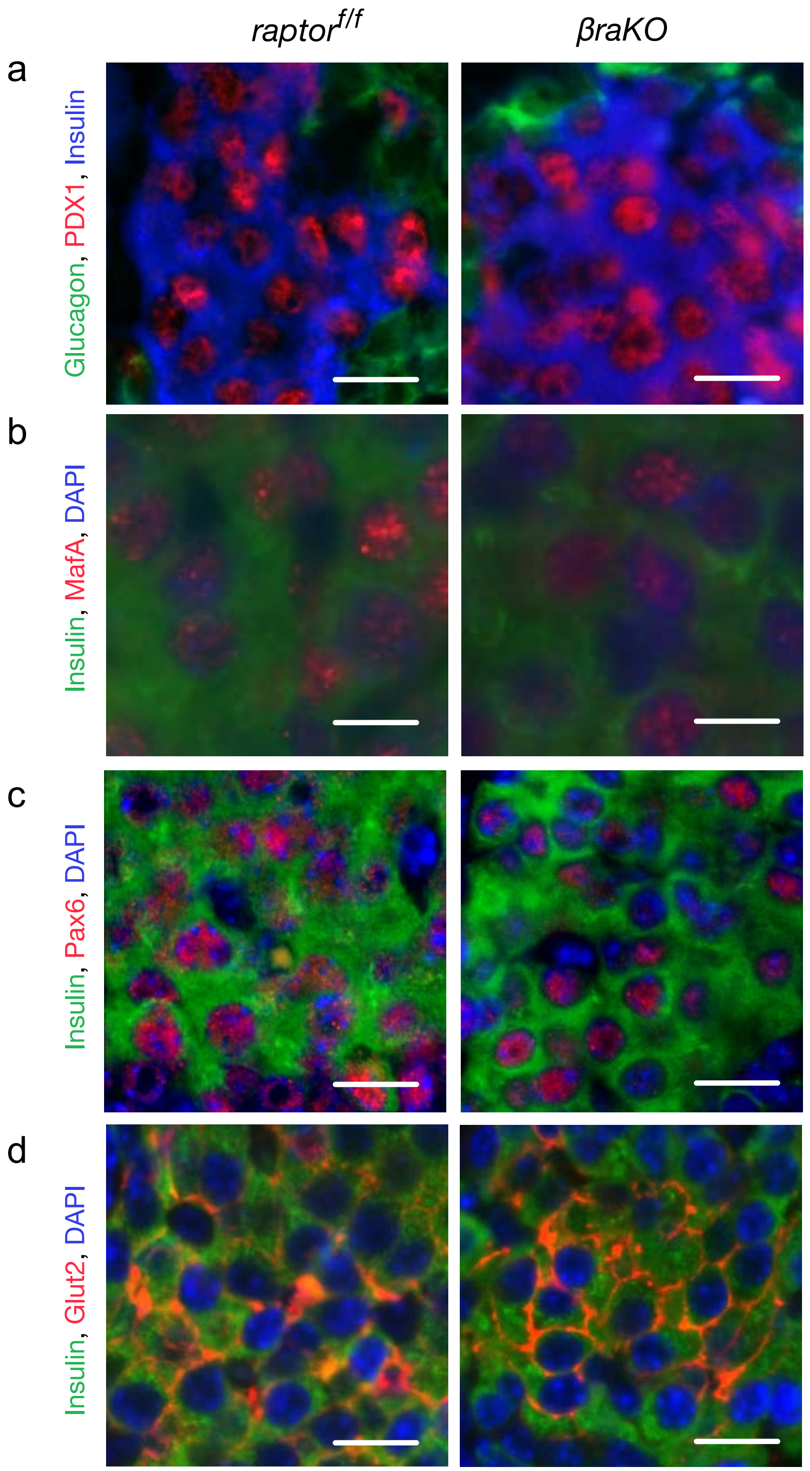

Supplementary Figure 6. Markers of β-cell maturation in *raptor*$^{f/f}$ and *βraKO* mice at 30 days of age.

Immunostaining for (a) insulin (blue), glucagon (green) and PDX1 (red). DAPI (blue), insulin (green) and (b) MafA (red), (c) Pax6 (red) and (d) Glut2 (red) in *raptor*$^{f/f}$ and *βraKO* mice at 30 days of age. Scale bar 18 μM (a,c,d) and 9 μM (b). Images are representative of 3 mice per group.

Supplementary Figure 7

90 days

β-catenin, Insulin, DAPI

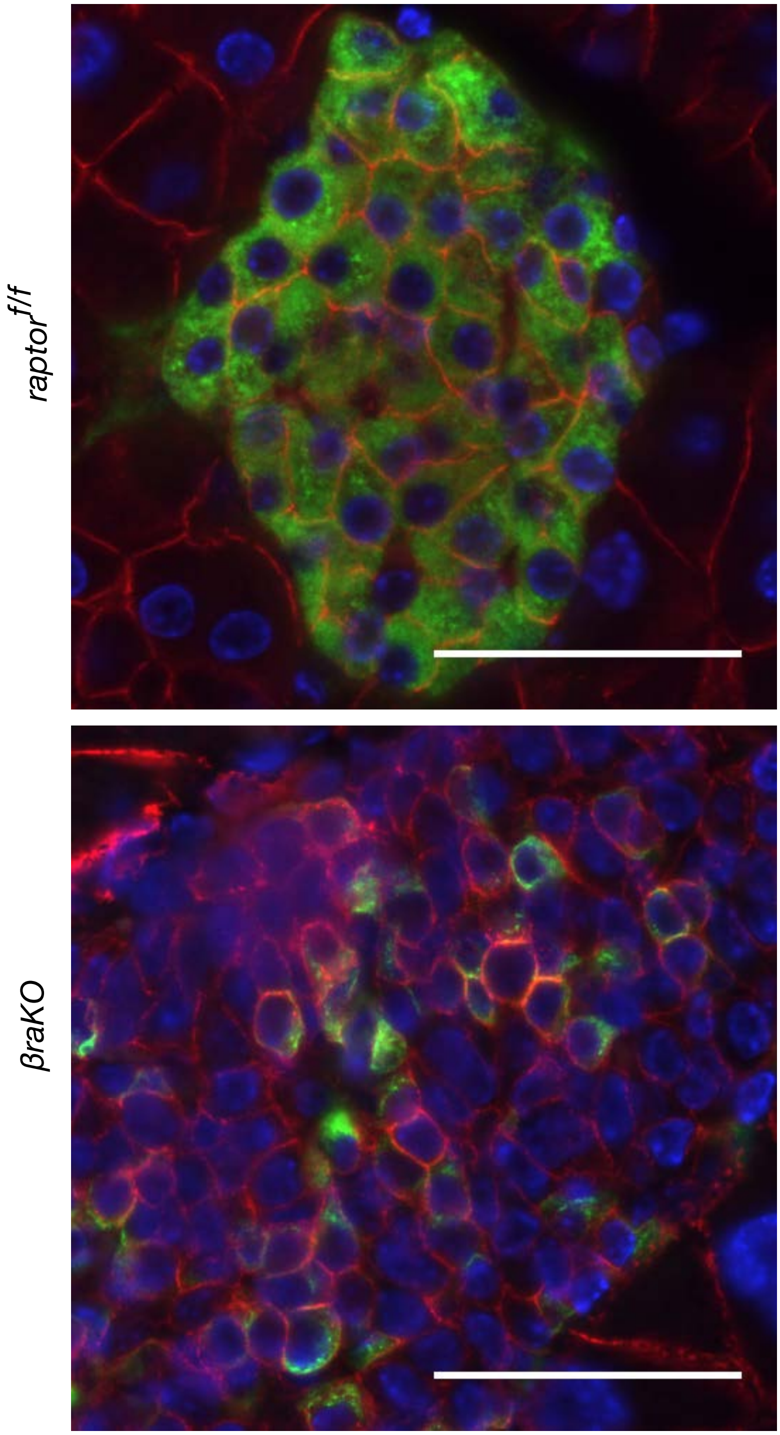

Supplementary Figure 7. Staining for β-catenin to analyze cell size in *raptor*$^{f/f}$ and *βraKO* mice at 90 days of age.

Cell size analysis in sections from 90 day-old mice stained for insulin (green), β-catenin (red) and DAPI (blue). Scale bar 50 μM. Images are representative of 5 mice per group.

Supplementary Figure 8

a

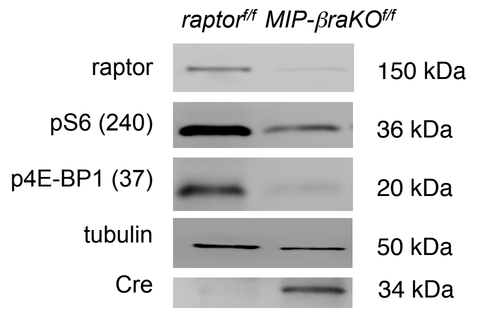

b

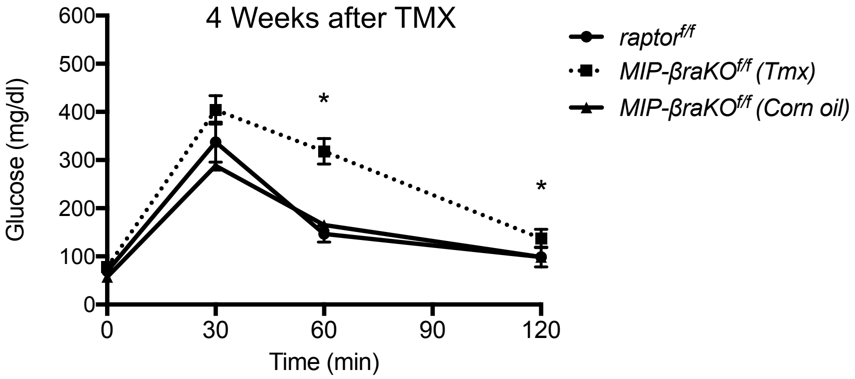

c

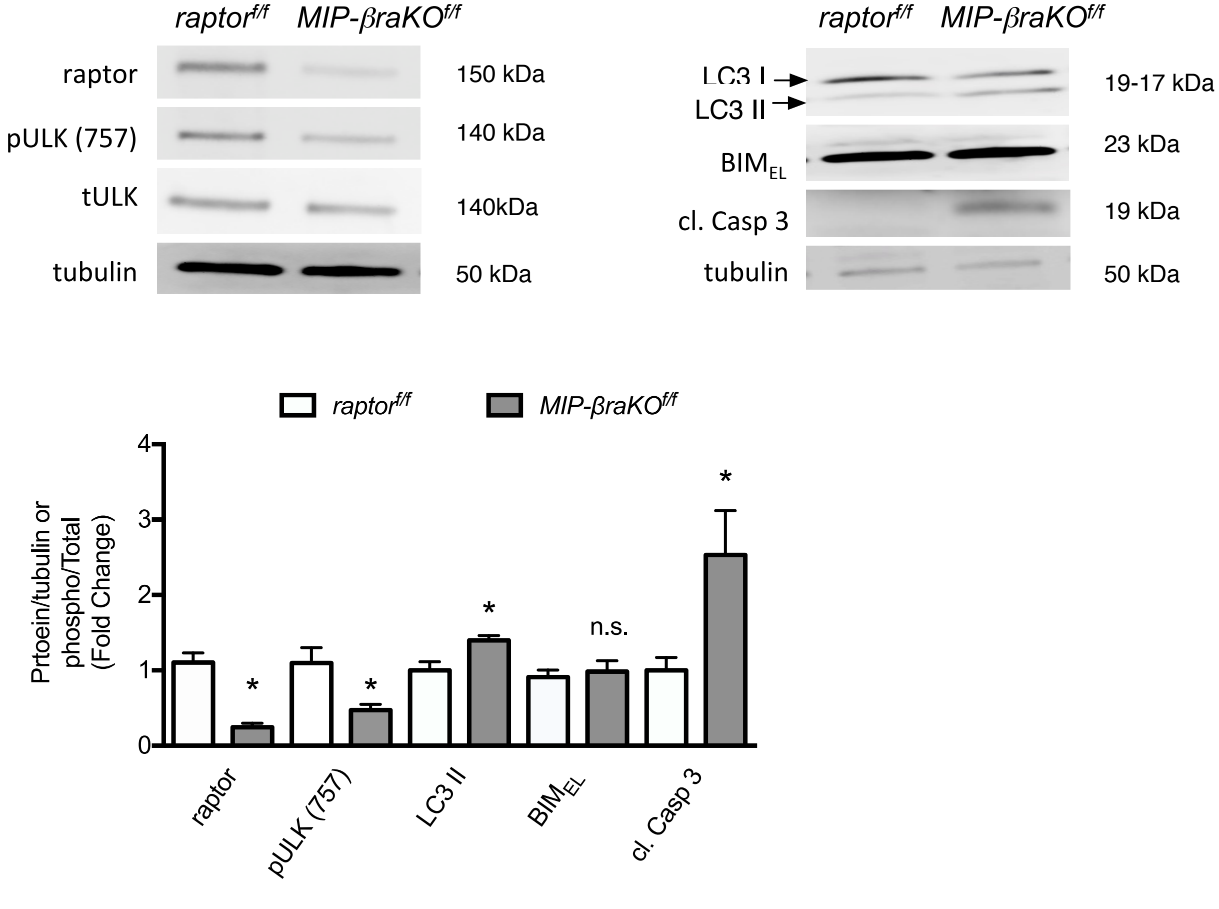

Supplementary Figure 8. Inducible Raptor deletion in β-cells results in impaired glucose tolerance and increased autophagic markers.

(a) Assessment of mTORC1 targets by immunoblotting using antibodies for raptor, pS6 (S240), p4E-BP1 (T37), Cre and tubulin in islet lysates from *raptor*$^{f/f}$ and *MIP-βraKO*$^{f/f}$ mice after 4 weeks of TMX injection (TMX administered at 2 months of age) (*n*=4). (b) Intraperitoneal glucose tolerance test after 4 weeks post-TMX injection (TMX administered at 2 months of age). *MIP-βraKO*$^{f/f}$ mice injected with corn oil are shown in these experiments as controls (*n*=4). (c) Immunoblotting and quantification for raptor, pULK (S757), total ULK and tubulin (left panel), LC3-I (upper band) and LC3-II (autophagosomal marker) (lower band), Bim_{EL}, cleaved-Caspase 3 and tubulin (right panel) after 2 weeks of TMX injection of *MIP-βraKO*$^{f/f}$ mice (10 weeks of age). A representative image from four independent experiments is included and each lane shows the expression levels from one mouse. Data expressed as means ± s.e.m., n.s. not significant, *$P < 0.05$. Nonparametric U test (Mann-Whitney).

Supplementary Figure 9

a

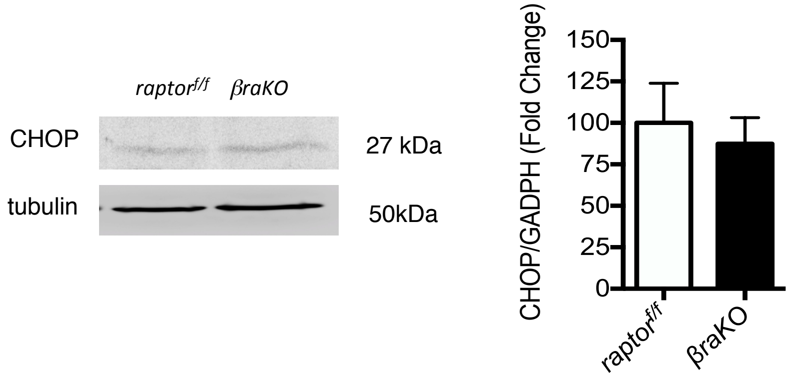

b

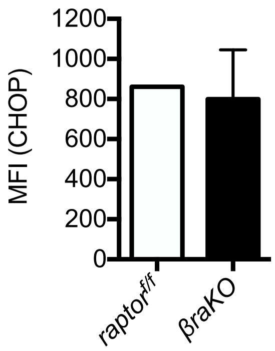

c

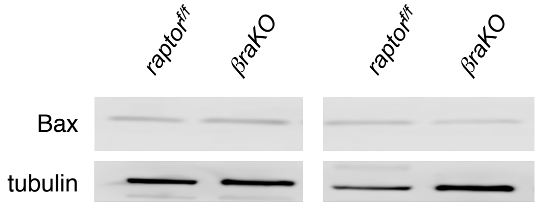

Supplementary Figure 9. Assessment of CHOP and Bax in control and *βraKO* mice. (a) Assessments of CHOP levels by immunoblotting in islets and (b) by flow cytometry in dispersed islets from 30 day-old *raptor*$^{f/f}$ and *βraKO* mice (Median Fluorescence Intensity, MFI). Data expressed as means ± s.e.m. Nonparametric U test (Mann-Whitney). (c) Immunoblotting for Bax and tubulin in *raptor*$^{f/f}$, *βraKO*, *βraKO;Eif4ebp2*$^{-/-}$ and *βraKO;caS6K;Eif4ebp2*$^{-/-}$ mice. A representative gel is shown in the Figure and each lane shows the expression levels from one mouse (*n*=3).

Supplementary Figure 10

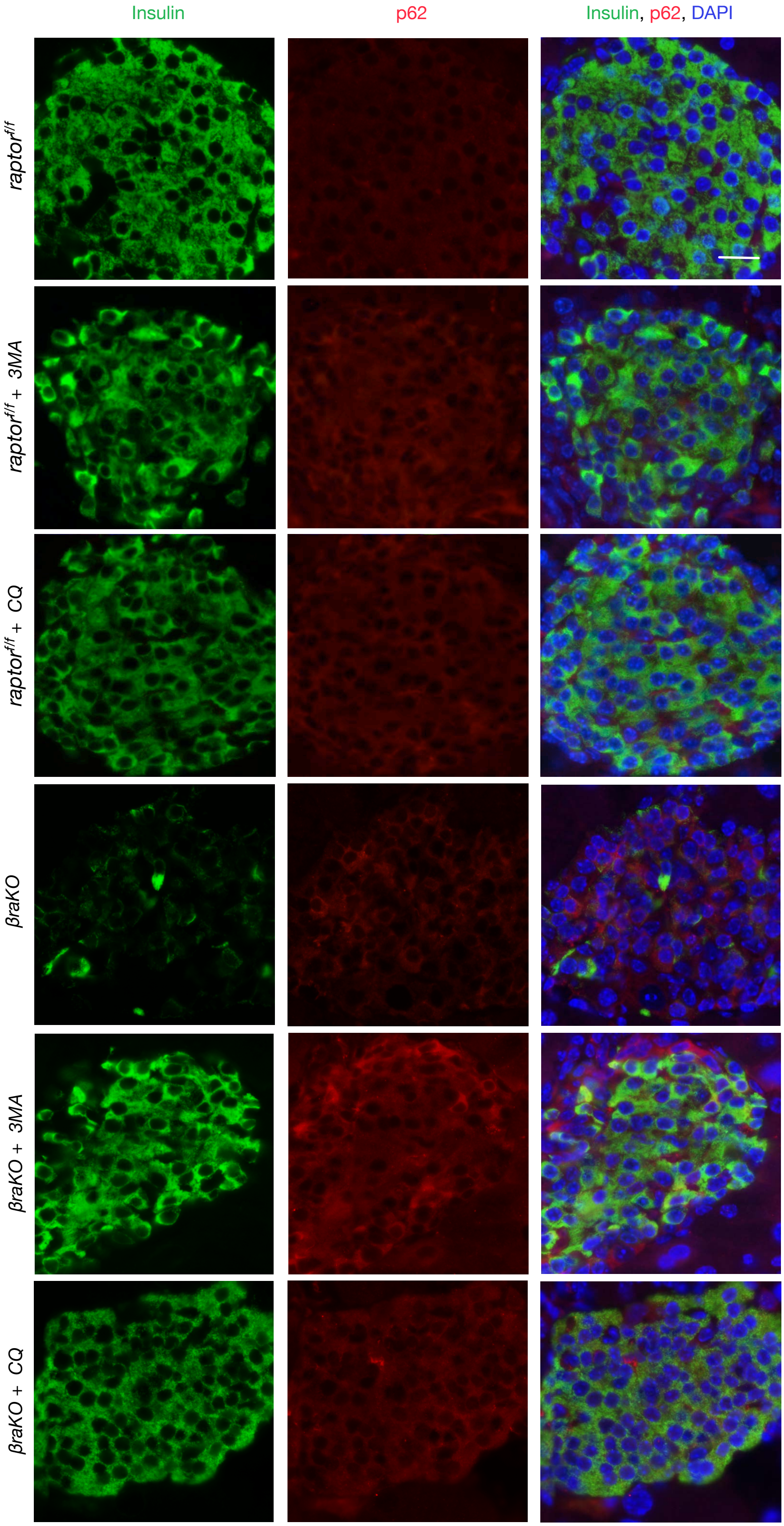

Supplemental Figure 10. p62 staining in control and *βraKO* mice treated with vehicle control or CQ and 3MA.

Immunostaining for insulin (green), p62 (red) and DAPI (blue) in sections from 80 day-old *raptor*$^{f/f}$ and *βraKO* mice treated for eight weeks with 3MA (15 mg/kg in 0.9% saline), CQ (7 mg/kg in 0.9% saline) or vehicle (saline). Treatment started in 18 day-old mice. Scale bar 20 μM

Supplementary Figure 11

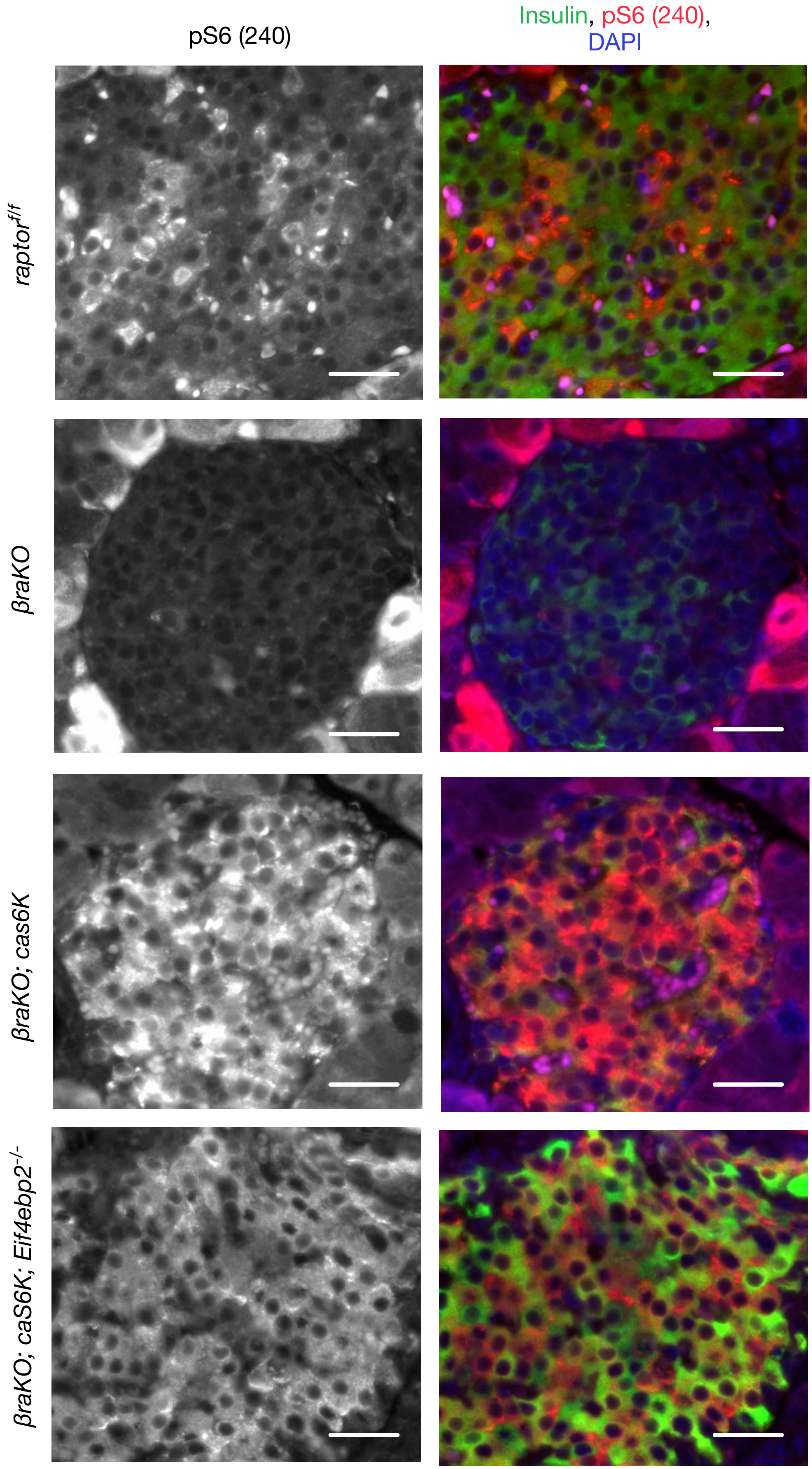

Supplementary Figure 11. Phosphorylation of S6 in control, *βraKO* mice and *βraKO* mice with gain of S6K function.

Immunostaining for pS6 (S240) (white, left panels and red, right panels), insulin (green), and DAPI (blue) in *raptor*$^{f/f}$, *βraKO*, *βraKO;caS6K and βraKO;caS6K;Eif4ebp2*$^{-/-}$ mice at 60 days of age. Scale bar 50 μM. Images are representative of 3 mice.

Supplementary Figure 12

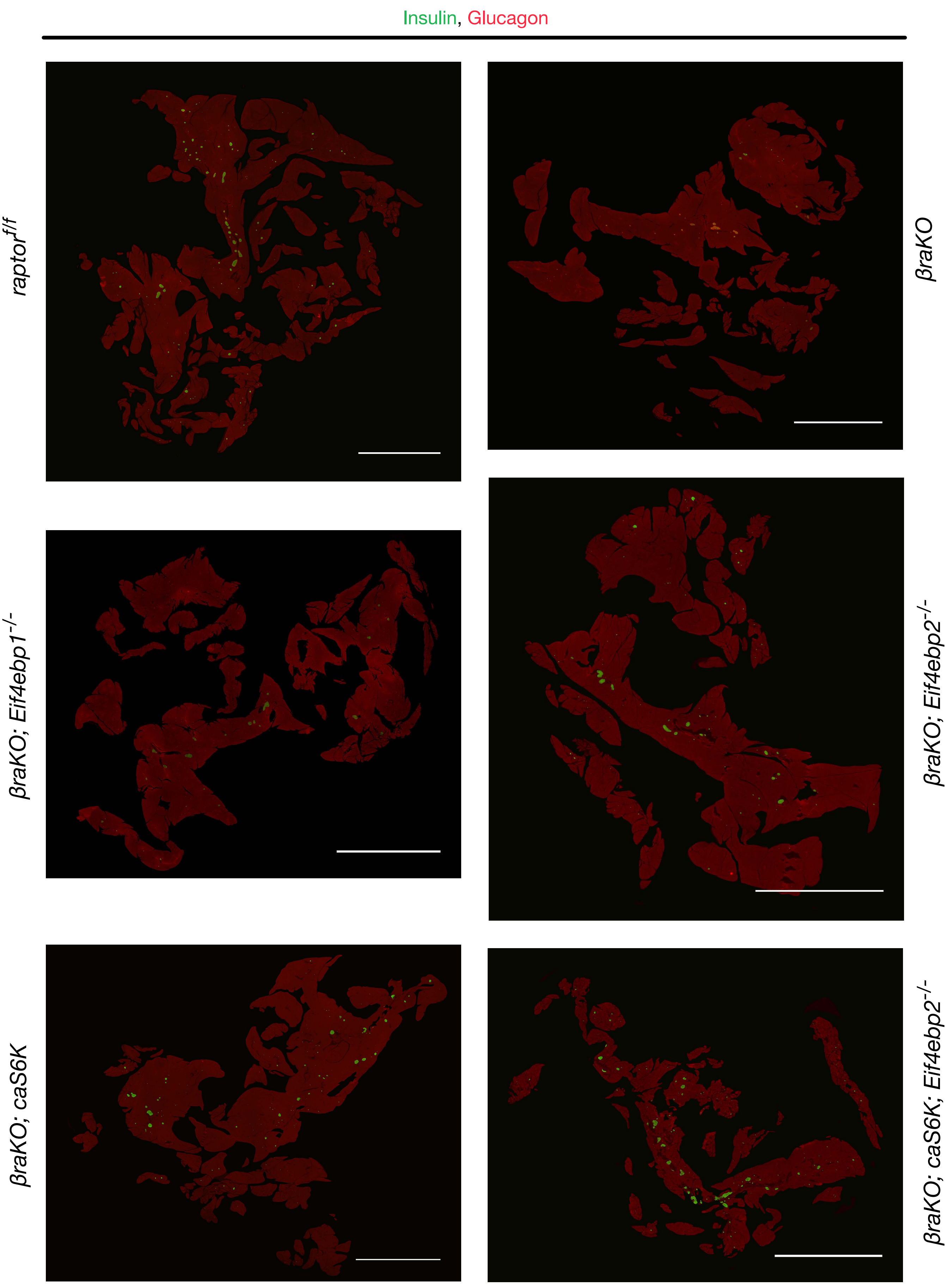

Supplementary Figure 12. Panoramic pictures of pancreas from control, *βraKO* and *βraKO* mice with gain of S6K and/or 4E-BP/eIF4E function.

Pancreatic sections from different experimental groups were stained for insulin (green) and glucagon (red). Exocrine pancreas can be distinguished by the red background. Scale bar 100 μM. Images are representative of 6 mice per group.

Supplementary Figure 13

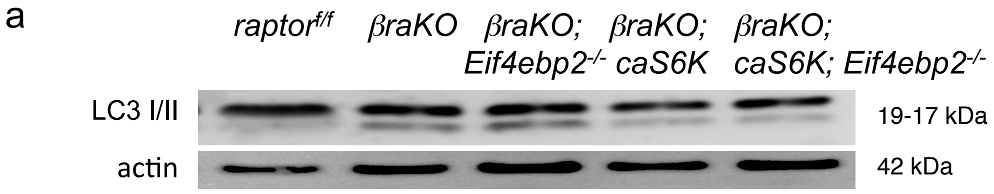

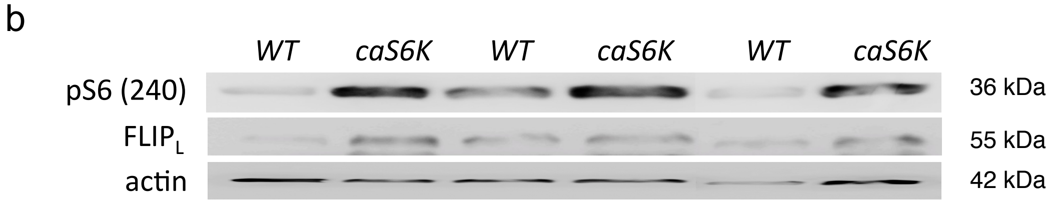

Supplementary Figure 13. Assessment of autophagy in mice with S6K overactivation and evaluation of FLIP$_L$ in *caS6K* mice.

(a) Immunoblotting for LC3 I/II (upper/lower band) and actin in different mouse models. A representative image from four independent experiments is included and each lane shows the expression levels from one mouse. (b) Immunoblotting for pS6 (S240), FLIP$_L$ and actin in islets from mice with overactivation of S6K *(caS6k)* and control (*WT*). A representative image from four independent experiments is included and each lane shows the expression levels from one mouse.

Supplementary Figure 14

a

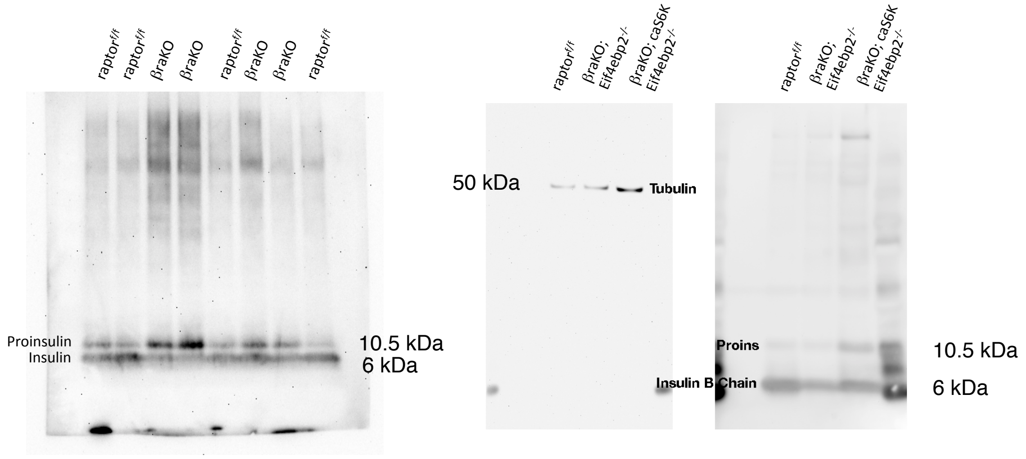

b

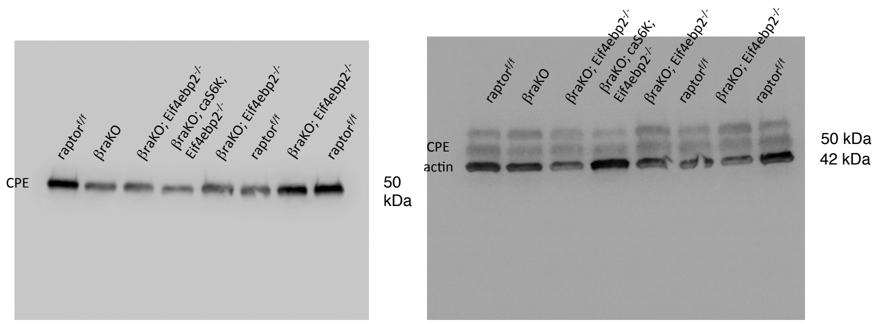

c

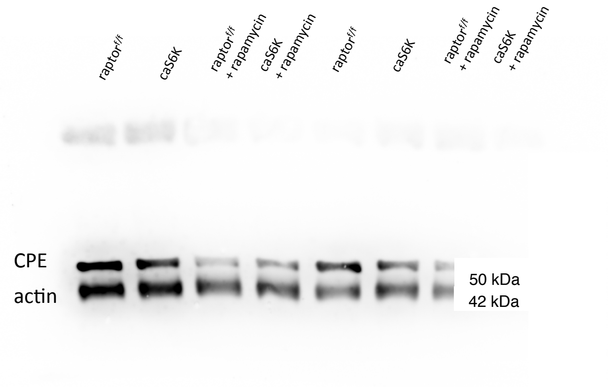

Supplementary Figure 14. Uncropped images of immunoblotting for proinsulin/insulin and CPE/actin and staining for CPE in control and *βraKO;caS6K;Eif4ebp2*$^{-/-}$ mice.

(a) Uncropped image of the blot for proinsulin/insulin in *raptor*$^{f/f}$ and *βraKO* mice (left) and *raptor*$^{f/f}$, *βraKO;Eif4ebp2*$^{-/-}$ *and βraKO;cas6K; Eif4ebp2*$^{-/-}$ mice. (b) Image of the immunoblotting for CPE (50 kDa) and actin (42 kDa) in *raptor*$^{f/f}$*, βraKO* and different mutant mice. (c) Immunoblotting for CPE (50 kDa) and actin (42 kDa) in *raptor*$^{f/f}$*, cas6K* treated or not with rapamycin.

Supplementary Figure 15

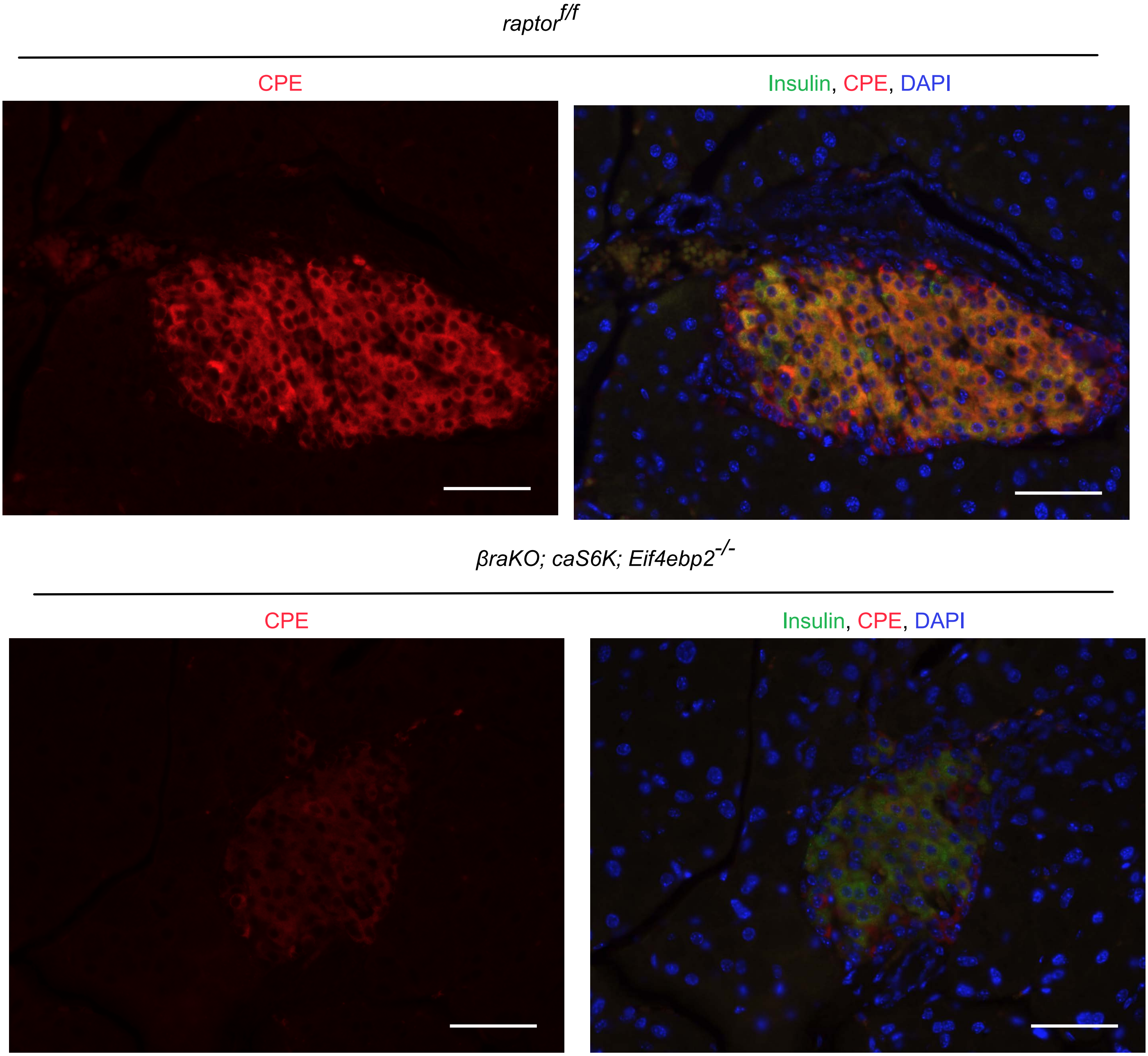

Supplementary Figure 15. Immunostaining for CPE and insulin in control and *βraKO;caS6K;Eif4ebp2*$^{-/-}$ mice.

Immunostaining for CPE (red), insulin (green) and counterstained with DAPI (blue) in *βraKO;caS6K;Eif4ebp2*$^{-/-}$ and control pancreas section (60 days of age). Scale bar 50 μM. Images are representative of 3 mice per group.

Supplementary Figure 16

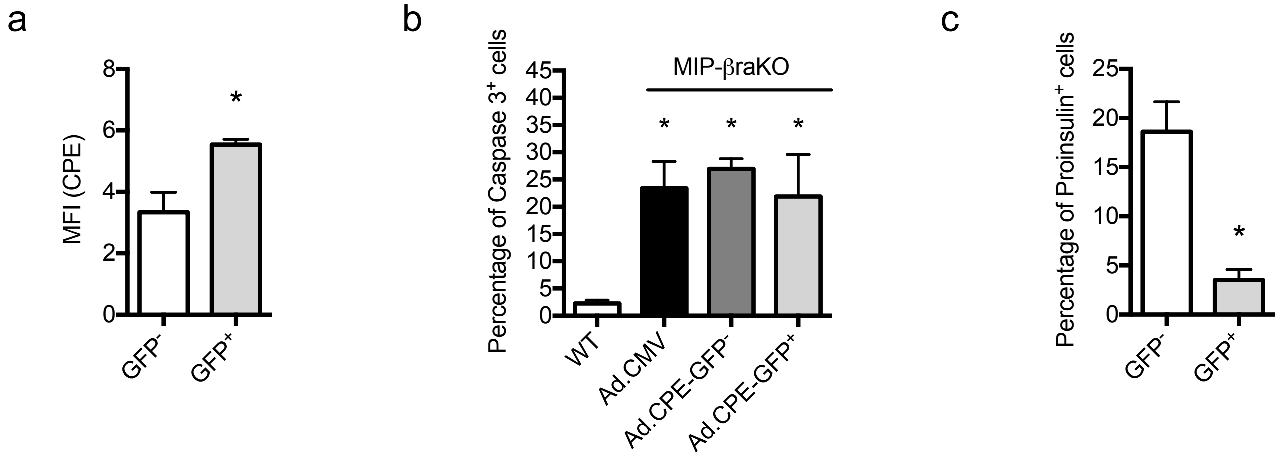

Supplementary Figure 16. Effects of reconstitution of CPE in *MIP-βraKO$^{f/f}$*.

(a) Flow cytometry analysis of CPE levels by MFI in β-cells from *MIP-βraKO$^{f/f}$* mice at 90 days of age (4 weeks after TMX injection) transfected (GFP positive) or not (GFP negative) with an adenovirus overexpressing CPE-GFP (*n*=4). (b) Percentage of active Caspase 3 positive in β-cells from WT and *MIP-βraKO$^{f/f}$* islets infected with a control (Ad. CMV) or a CPE-GFP adenovirus (Ad. CPE-GFP^{+}). CPE-GFP^{-} cells were cells non-infected by the CPE-GFP adenovirus (*n*=4). (c) Flow cytometry analysis for proinsulin in β-cells using the same conditions as described for Supplemental Fig. 13a. Data expressed as means ± s.e.m., $^{*}P < 0.05$. Nonparametric U test (Mann-Whitney).

Supplementary Figure 17

a

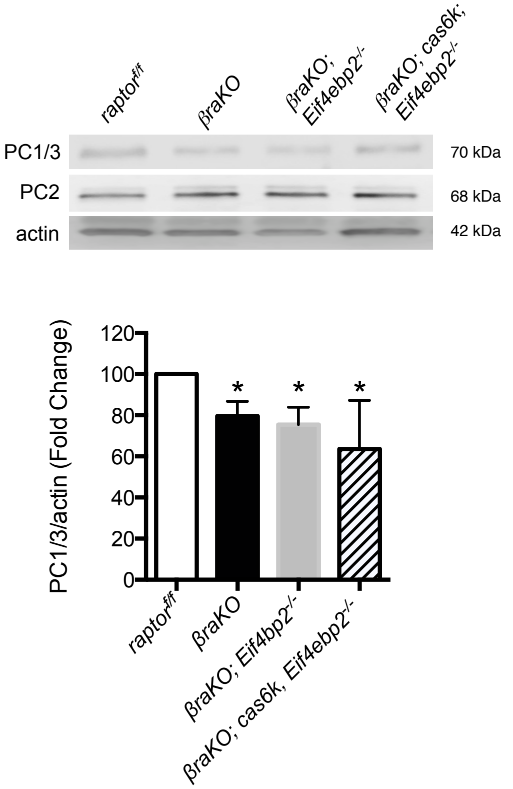

b

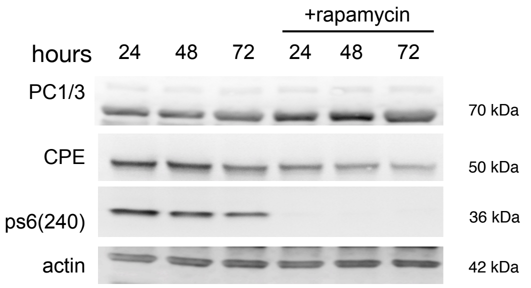

Supplementary Figure 17. Assessment of PC1/3 and PC2 in islets from *βraKO*, *βraKO*;*Eif4ebp2*$^{-/-}$, *βraKO;caS6K;Eif4ebp2*$^{-/-}$ and in MIN6 cells after rapamycin treatment.

(A) Immunoblotting and quantifications for PC1/3, PC2 and actin in *βraKO*, *βraKO*;*Eif4ebp2*$^{-/-}$, *βraKO;caS6K;Eif4ebp2*$^{-/-}$ and control mice (*n*=4). (B) Immunoblotting for PC1/3, CPE, pS6 (S240) and actin in MIN6 cells treated or not with rapamycin for 24, 48 or 72 hours. A representative image from three independent experiments is included. Data expressed as means ± s.e.m., *P < 0.05. Nonparametric U test (Mann-Whitney).

Supplementary Figure 18

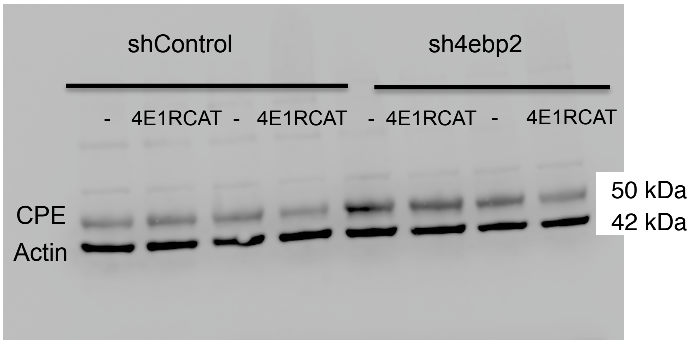

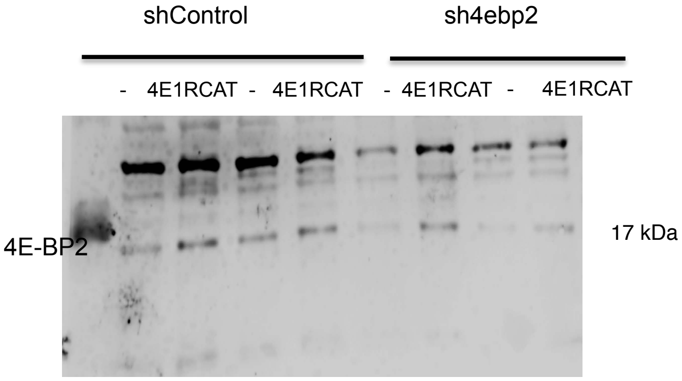

Supplementary Figure 18. Uncropped images of CPE/actin immunoblotting in MIN6 control or 4E-BP2 silencing (sh4ebp2).

(a) Uncropped image of immunoblotting for CPE (50 kDa) and actin (42 kDa) (upper image) and 4E-BP2 (lower image) in control MIN6 cells or MIN6 cells with stable silencing of 4E-BP2 (sh4ebp2) treated or not with 4E1RCat (25 μm) for 24h.

Supplementary Figure 19

RNA Folding of Mouse CPE UTR.seq

Min.Energy: -20.06 Temperature: 37

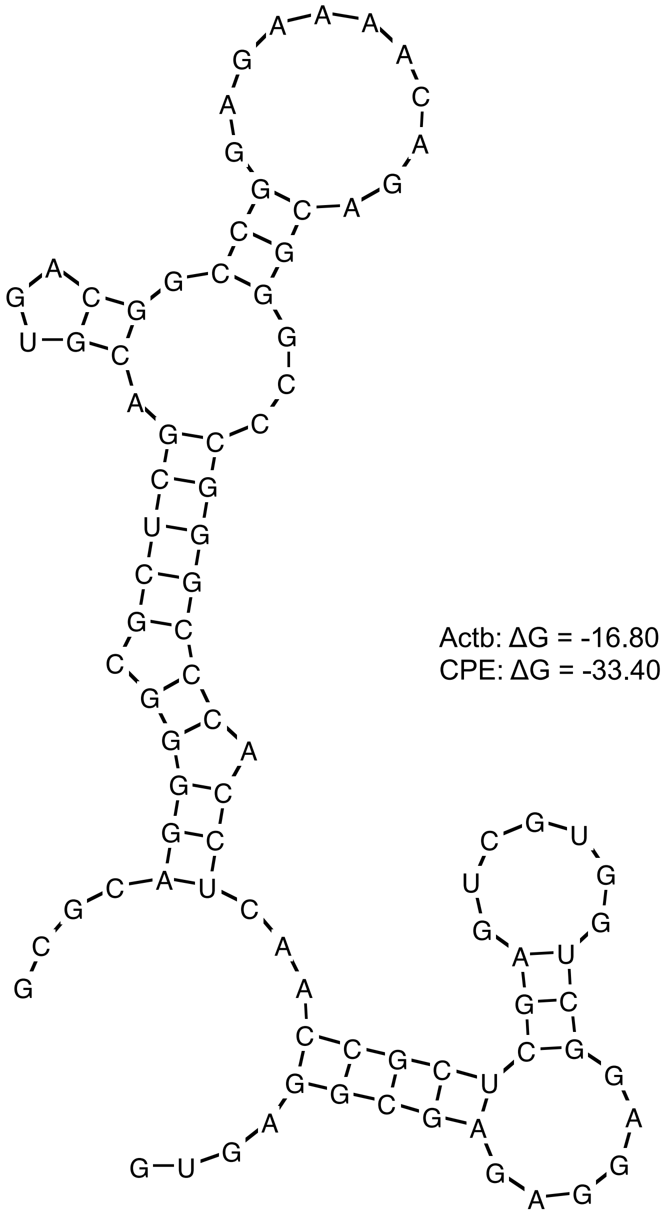

Actb: ΔG = -16.80 kcal/mol
CPE: ΔG = -33.40 kcal/mol

Mouse CPE
GC Content: 71.4
UTR length: 91

GTGAGGCGAGAGGAGGCTGGTGC
TGAGCTCGCCAACTCCACCCGGG
CCCGGGCAGACAAAAGAGGCCGG
C AGTGCAGCTCGCGGGGACGCG

Supplementary Figure 19. Analysis of the secondary structure of the 5' UTR region of CPE.

RNA secondary structure of 5'-UTR of CPE mRNA. Secondary structure determined by GeneQuest 12.1 (Part of the DNASTAR LaserGene suite. DNASTAR Inc., Madison WI, USA).

Supplementary Figure 20

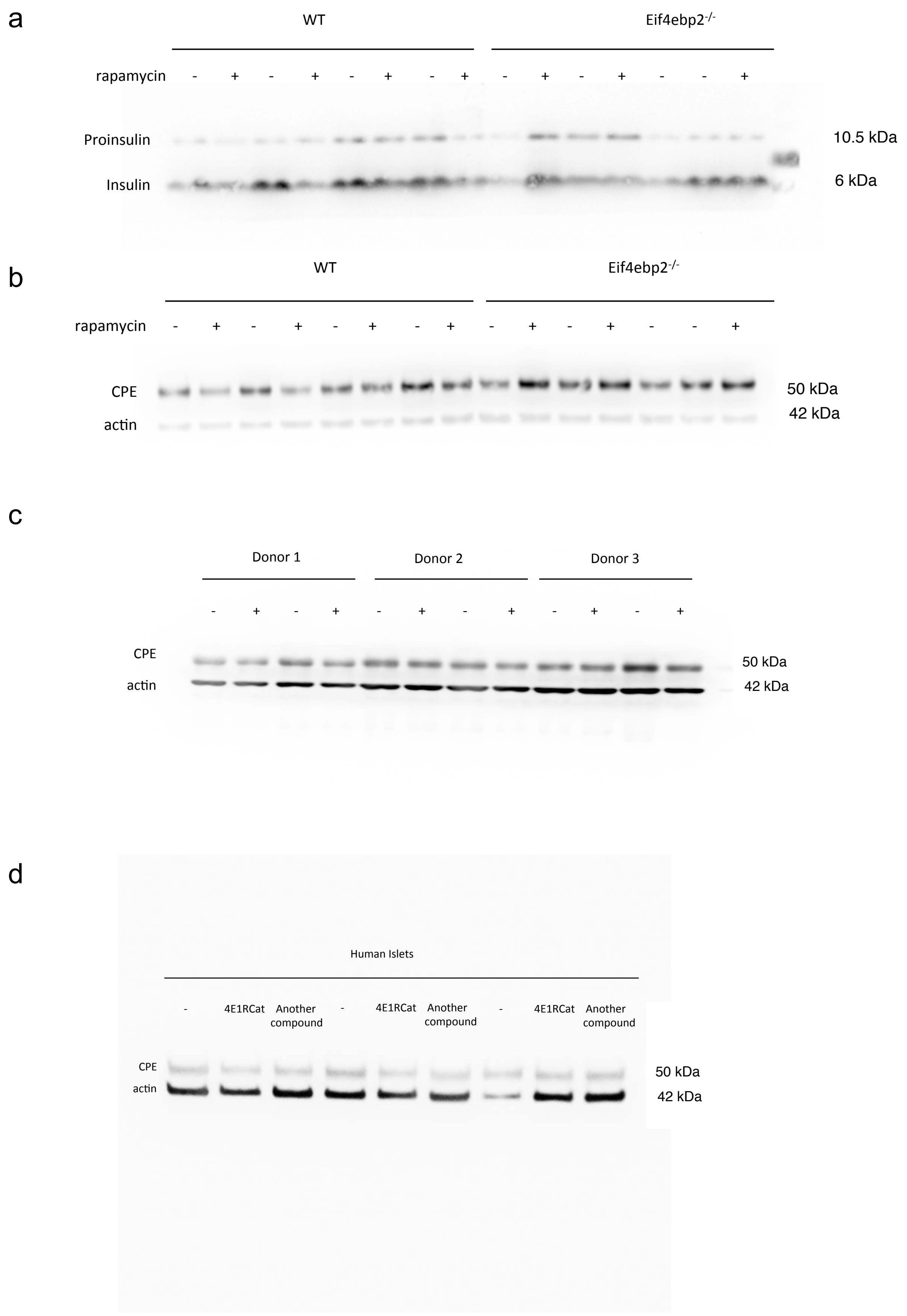

Supplementary Figure 20. Uncropped images of immunoblotting for proinsulin/insulin and CPE/actin in mice and human islets treated with rapamycin or 4E1RCat.

(a,b) Uncropped blot for proinsulin/insulin, CPE (50 kDa) and actin (42 kDa) in *WT and Eif4ebp2* mice treated or not with rapamycin intraperitoneally. (c) Uncropped western blot for CPE (50 kDa) and actin (42 kDa) in human islets from three different donors treated with rapamycin or vehicle control. (d) Uncropped image of the immunoblotting for CPE (50 kDa) and actin (42 kDa) in human islets from three different donors treated with 4E1RCat (25 μm) or vehicle control (islets were also treated with "another unrelated compound" not included in this manuscript) for 24h.

Supplementary Figure 21

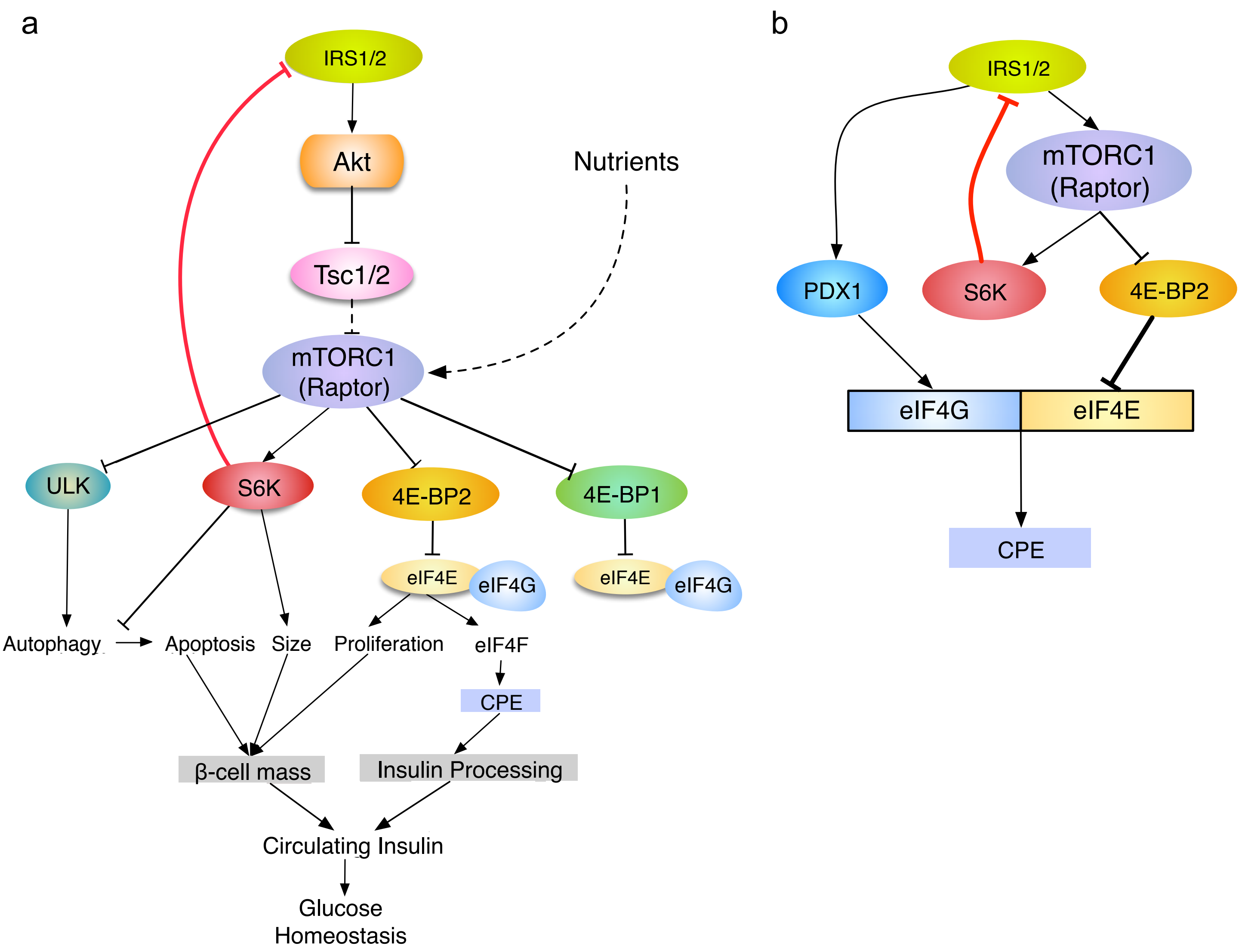

Supplementary Figure 21. Pathways downstream of mTORC1/Raptor and their effects.

(a) Schematic summarizes the results of the current experiments by showing how mTORC1 acting in downstream targets regulates β-cell mass and insulin processing. Activation of insulin and growth factor receptors recruit insulin receptor substrates (IRS) and induce phosphorylation of AKT. TSC2 phosphorylation and inactivation by Akt releases the inhibition of Rheb, leading to activation of mTORC1. mTORC1 regulates many biological processes by phosphorylation of many downstream. The current studies propose the following model in β-cells: 1. Phosphorylation of ULK regulates autophagy and β-cell survival, 2. Activation of mTORC1/S6K axis inhibits insulin signaling by a negative feedback loop mediated by phosphorylation and degradation of IRS1/2. In addition, mTORC1/S6K regulates cell size, inhibits autophagy and promotes survival. 3. mTORC1 phosphorylates 4E-BP1 and 2 leading to dissociation of eIF4E and initiates cap-dependent translation of mRNAs with complex 5'UTR structures. The current work suggests that 4E-BP2/eIF4E (and not 4E-BP1) regulates CPE levels and controls insulin processing. In addition, we recently demonstrated that the 4E-BP2/eIF4E axis also modulates β-cell proliferation [27]. (b) The current diagram shows that insulin receptor signaling regulates insulin processing by two distinct pathways that converge on the formation and activation of the eIF4G:eIF4E and CPE translation. These two parallel pathways include: 1. An insulin/IRS1/2/PDX1 axis that regulates eIF4G transcription. This axis can be negatively regulated by the negative feedback on IRS1/2 signaling induced by S6K overactivation, and 2. A second pathway uncovered in the current studies is mediated by 4E-BP2 controlling the availability of eIF4E to form the

eIF4E:eIF4G complex. In the current model, S6K overactivation neutralizes the effect of 4E-BP2 on CPE and proinsulin processing by reducing IRS1/2/PDX1 and ultimately eIF4G levels. The importance of these two parallel pathways on CPE levels and insulin processing during normal conditions or diabetogenic conditions is unclear but these findings underscore the importance of insulin signaling on controlling insulin processing.

Supplementary Table 1. Islet donors

**Donor**	**1**	**2**	**3**	**4**	**5**
Age (years)	63	15	50	50	30
Sex (male/female)	Male	Male	Male	Female	Male
Race	White	White	Black	White	Hawaiian
BMI (kg/m^2)	38.6	23	31.7	28.50	33
Weight (kg)	118.7	75	103	80	110
Height (inches)	69	71	71	66	72
Cause of death	Anoxia	Head trauma	Head trauma	Stroke	Anoxia
T2 Diabetic donor status	No	No	No	No	No
Post culture viability (%)	98	94	97	95	95
Post culture purity (%)	70	90	85	90	90

Supplementary Table 2. Antibodies

Antibody	Specie	Source	Concentration	Catalog
4E-BP1	Rabbit	Cell Signaling	1:1000	9644
4E-BP2	Rabbit	Cell Signaling	1:1000	2845
Actin	Mouse	Sigma	1:4000	A5441
Active caspase 3 BV650	Rabbit	BD Trans.	5 μl per test	564096
Bax	Rabbit	Cell Signaling	1:1000	2772
Bim	Rabbit	Cell Signaling	1:1000	2819
Caspase 3, cleaved	Rabbit	Cell Signaling	1:1000	9661
CHOP	Rabbit	Santa Cruz	1:500	SC-575
CPE	Mouse	BD Trans.	1:3000	610758
Eif4GI	Rabbit	Cell Signaling	1:1000	8701
Flip	Mouse	Santa Cruz	1:1000	sc-5276
Glucagon	Mouse	Abcam	1:1000	ab10988
Glut2	Rabbit	Chemicon	1:400	AB1662
Insulin	Guinea Pig	Dako	1:400	A0564
Insulin	Mouse	Sigma	1:400	I2018
Insulin APC-Conjugated	Rat	R&D Systems	1 μl per test	IC1417A
IRS2	Rabbit	Cell Signaling	1:1000	3089
Ki67	Rabbit	Vector	1:200	NCL-Ki67p/VP-K451
LC3-I/II	Rabbit	Novus Biologicals	1:1000	NB100-2220

MAfA	Rabbit	Bethyl Lab	1:100	00352
MSH	Rabbit	M. Low's Lab		
p-4E-BP1 (T37)	Rabbit	Cell Signaling	1:1000	9459
p-S6 (S235)	Rabbit	Cell Signaling	1:1000	4858
p-S6(S240)	Rabbit	Cell Signaling	1:1000	5364
p-ULK (S757)	Rabbit	Cell Signaling	1:1000	6888
p62	Rabbit	Cell Signaling	1:1000	8025
Pancreatic Polypeptide	Guinea Pig	Millipore	1:50	4041-01
Pax6	Rabbit	Covance	1:300	PRB-278P
PC1/3	Rabbit	Lindberg Lab	1:1000	
PC2	Rabbit	Lindberg Lab	1:1000	
PDX1	Rabbit	Millipore	1:800	07-696
POMC	Rabbit	Phoenix Ph.	1:200	
Proinsulin	Mouse	P. Arvan's Lab	1:500	-
Proinsulin MAb (253627)	Mouse	R&D Systems	1 μl per test or 1:500	MAB13361-SP
Raptor	Rabbit	Cell Signaling	1:1000	4978
S6	Rabbit	Cell Signaling	1:1000	2317
Somatostatin	Rabbit	Santa Cruz	1:400	sc-13099
Synaptophysin	Rabbit	Abcam	1:200	ab32127
Tubulin	Mouse	Sigma	1:3000	T 5168
ULK	Rabbit	Cell Signaling	1:1000	8054
β-catenin	Mouse	Sigma	1:1000	610153
